# Development and validation of a predictive model for short-term symptom relief after organophosphate poisoning

**DOI:** 10.3389/fmed.2026.1637549

**Published:** 2026-04-28

**Authors:** Guoliang Huang, Shaobo Zhang, Binbin Wu, Zuxiong Su, Bingwen Li, Po Mao, Shuanghui Hu, Jiangping Li

**Affiliations:** Emergency Center, Anxi County Hospital, Quanzhou, Fujian, China

**Keywords:** cholinesterase activity, Glasgow Coma Scale, LASSO regression, nomogram, organophosphate poisoning, outcomes, symptom resolution

## Abstract

**Background:**

Acute organophosphate (OP) poisoning outcomes vary significantly despite standard atropine/pralidoxime (PAM) therapy. Early identification of patients likely to improve rapidly could optimize resource allocation and individualized care.

**Methods:**

This retrospective study analyzed 138 adult acute OP poisoning cases (2018–2023). Patients were randomly split into training (*n* = 96) and validation (*n* = 42) cohorts. LASSO regression analyzed 14 potential predictors (demographics, clinical presentation, labs) to select features with non-zero coefficients at the 1-SE *λ* threshold. These were used in multivariable logistic regression to develop a nomogram predicting clinical improvement (any reduction in the poisoning symptom score (PSS) within 48 h post-admission). Model validation included discrimination (ROC/AUC), calibration [calibration plots and mean absolute error (MAE)], and clinical utility (decision curve analysis, DCA).

**Results:**

Four independent predictors were identified: baseline PSS, admission serum cholinesterase, admission Glasgow Coma Scale (GCS) score, and major comorbidities. The nomogram showed strong discrimination (optimism-corrected AUC 0.930 in the training cohort; AUC 0.905 in the validation cohort) and good calibration (MAE 0.034 and 0.066). Decision-curve analysis showed positive net benefit over treat-all and treat-none across clinically relevant thresholds.

**Conclusion:**

We developed and validated a practical nomogram predicting 48-h symptom relief in acute OP poisoning. This tool can enhance early risk stratification, guide clinical decisions, and support efficient ICU resource utilization.

## 1 Introduction

Acute organophosphate (OP) poisoning remains a major contributor to global morbidity and mortality, particularly in agricultural regions of Asia and Africa, where it accounts for an estimated 300,000 deaths annually from pesticide self-poisoning (1). OP compounds irreversibly inhibit acetylcholinesterase, precipitating a cholinergic crisis characterized by muscarinic, nicotinic, and central nervous system manifestations (2). Despite early administration of atropine and pralidoxime (PAM), clinical outcomes vary widely, and prolonged intensive-care treatment is often required (3, 4). In this context, early, individualized risk stratification is clinically important to inform triage decisions, monitoring intensity, and timely escalation of supportive therapy.

Composite bedside severity frameworks are commonly used to grade acute poisoning and summarize the overall burden of organ dysfunction (5, 6). In the present study, we used a poisoning symptom score (PSS) derived from five clinically relevant domains—gastrointestinal, respiratory, cardiovascular, central nervous system, and metabolic/general manifestations—to quantify symptom burden at admission and again at 48 h. In OP poisoning, higher baseline symptom burden is associated with worse short-term clinical evolution, yet isolated markers such as age, serum cholinesterase activity, or Glasgow Coma Scale (GCS) score are often interpreted separately and therefore provide limited bedside discrimination when used alone (2, 7–9). Accordingly, there is a practical need for an integrative prediction approach that combines routinely available variables into an individualized probability estimate of early clinical improvement.

Penalized regression techniques such as the least absolute shrinkage and selection operator (LASSO) can address multicollinearity and overfitting by simultaneously performing variable selection and coefficient shrinkage, thus enhancing model stability in clinical datasets with multiple correlated predictors (10). Beyond model development, nomograms provide a clinically intuitive implementation of multivariable prediction by transforming regression coefficients into a points-based graphical interface, enabling clinicians to compute patient-specific risk estimates quickly at the bedside (11–13). Importantly, nomogram-based models have gained increasing attention in acute poisoning research because early disposition and supportive-care decisions (e.g., ICU admission, airway management, and cardiotoxicity monitoring) often must be made under uncertainty. Recent studies have developed and validated nomograms to support ICU disposition in acute clozapine intoxication (14), to predict the need for mechanical ventilation among acutely intoxicated patients with impaired consciousness (15), and to estimate in-hospital adverse cardiovascular events in poisoning with cardiotoxic agents (16). Collectively, these works underscore the growing role of nomograms as practical clinical prediction tools in toxicology and highlight their potential to complement conventional severity scoring when individualized, outcome-specific prediction is needed. Guidelines such as TRIPOD emphasize transparent development and reporting of multivariable prediction models to ensure reproducibility and clinical utility (17, 18).

To our knowledge, no prior study has integrated LASSO-based selection with multivariable logistic regression to construct and validate a nomogram for predicting short-term symptom relief after acute OP poisoning. Accordingly, we conducted a retrospective cohort study of 138 patients, applied ten-fold cross-validated LASSO to 14 candidate predictors, refined the model via multivariable logistic regression, and developed a nomogram estimating the probability of PSS reduction at 48 h. We evaluated model discrimination, calibration, and clinical utility in both the training and validation cohorts, in accordance with TRIPOD recommendations.

## 2 Materials and methods

### 2.1 Study design and patient identification

This retrospective cohort study enrolled consecutive adult patients with acute organophosphate (OP) poisoning admitted to our tertiary care center between January 2018 and December 2023. The study protocol was approved by the Medical Ethics Committee of Anxi County Hospital, Quanzhou City, and all procedures were conducted in accordance with the Declaration of Helsinki.

To ensure reproducible case ascertainment, potential cases were first screened from the electronic health record (EHR) using the International Classification of Diseases, 10th revision (ICD-10) diagnosis code T60.0 (toxic effect of organophosphate and carbamate insecticides). When available in the EHR, pesticide-related external cause codes were also retrieved to characterize exposure intent, including accidental exposure (X48), intentional self-poisoning (X68), assault (X88), or undetermined intent (Y18).

Two investigators independently reviewed each screened record to confirm acute OP poisoning. A case was considered confirmed when exposure to an OP pesticide (ingestion, inhalation, or dermal contact) was documented and the treating team diagnosed OP poisoning based on a compatible cholinergic toxidrome, supported by at least one of the following: decreased serum cholinesterase activity on admission and/or identification of an OP agent (eg, container label or history from the patient/family). Patients whose final diagnosis after chart review indicated carbamate-only intoxication, non-pesticide poisoning, or a primary alternative diagnosis were excluded.

Inclusion criteria were: (1) age >= 18 years; (2) confirmed acute OP poisoning; (3) baseline poisoning symptom score (PSS) assessed on admission; and (4) repeat PSS assessment available at 48 h after admission. Exclusion criteria were: (1) incomplete medical records for key variables (baseline or 48-h PSS); (2) mixed or co-ingestion poisoning with other substances (e.g., paraquat, sedatives, corrosives); or (3) terminal comorbid conditions (e.g., end-stage malignancy) that precluded standard management and follow-up.

For split-sample internal validation, the eligible cohort was randomly allocated into a training cohort (70%, *n* = 96) and an internal validation cohort (30%, *n* = 42) using stratified random sampling to preserve the outcome proportion in each subset.

### 2.2 Sample size considerations

Because this was a retrospective study, the sample size was determined by the number of eligible admissions during the predefined study period. We evaluated the training cohort using an events-per-parameter framework. In the training cohort (*n* = 96), 45 patients achieved the primary outcome (ΔPSS > 0) and 51 did not. With four predictors ultimately retained in the final model, the effective sample size satisfied conventional minimum requirements for model development.

### 2.3 Data collection and candidate predictors

Demographic, clinical, and laboratory data were abstracted from the EHR using a standardized case report form. Fourteen candidate predictors were predefined based on clinical relevance and prior literature: age; sex; baseline PSS; route of exposure (oral, dermal, inhalation); estimated dose; time from exposure to hospital admission; Glasgow Coma Scale (GCS) score at admission; serum cholinesterase activity at admission; arterial lactate; arterial blood pH; time to first atropine dose; cumulative 24-h atropine dose; time to first pralidoxime (PAM) dose; and presence of major comorbidities.

Time to first PAM dose was calculated from the timestamp of emergency department triage registration to the start time of the initial pralidoxime infusion and recorded to the nearest minute. Major comorbidity was defined as the documented presence of at least one of the following chronic conditions: hypertension, diabetes mellitus, coronary artery disease, chronic obstructive pulmonary disease, chronic kidney disease, chronic liver disease, or active malignancy. Each patient was assigned a binary indicator (0 = none; 1 = at least one condition) for inclusion in the multivariable model. Because item-level data needed to compute the Charlson Comorbidity Index were incomplete in this retrospective cohort, this binary comorbidity variable was used as a pragmatic substitute.

### 2.4 Symptom assessment

Baseline PSS represented the total symptom-burden score recorded at admission (or within the first complete assessment window after arrival), and 48-h PSS was reassessed using the same framework 48 h after treatment initiation. The score comprised five domains—gastrointestinal, respiratory, cardiovascular, central nervous system, and metabolic/general manifestations—each graded from 0 to 4 according to the most severe documented findings at that time point (0 = none, 1 = mild, 2 = moderate, 3 = severe/life-threatening, 4 = death). The total PSS therefore ranged from 0 to 20. For descriptive symptom-incidence analyses, cholinergic and neuromuscular manifestations were also abstracted from physician and nursing documentation at admission as binary variables (0 = absent, 1 = present), including vomiting, diarrhea, sweating, chest crepitations, abdominal colic, muscle fasciculations, and muscle weakness.

### 2.5 Outcome definition

The primary outcome was short-term symptom relief, defined as any decrease in the PSS at 48 h post-admission compared with baseline (ΔPSS > 0). Patients with ΔPSS ≤0 were classified as non-relieved. This binary outcome (*Y* = 1 if relieved, 0 if not) was used for all subsequent analyses. To reduce confounding by initial illness burden, baseline PSS was included as a predictor in the multivariable models.

### 2.6 `treatment protocol

All patients were managed according to our institution’s standard atropine and PAM protocol. Briefly, upon diagnosis of OP poisoning, atropine was initiated with a 2 mg intravenous bolus and doubled every 5–10 min until atropinization endpoints (drying of bronchial secretions and heart rate >= 80 beats/min) were achieved, followed by continuous infusion at 0.05 mg/kg/h. Pralidoxime was administered as a 1 g intravenous loading dose over 30 min, then 1 g every 8 h for 48 h, together with supportive care as clinically indicated.

### 2.7 Complication classification

All complication events occurring after admission were systematically abstracted from the EHR by two independent investigators; discrepancies were resolved by consensus with a senior clinician. Complications were classified into two time windows. Short-term complications (<= 48 h) included respiratory failure requiring mechanical ventilation, clinically significant cardiac arrhythmias (eg, ventricular tachycardia/fibrillation), seizure clusters, and diffuse muscle fasciculations. Long-term complications (3–28 days) included intermediate syndrome (delayed-onset muscle weakness), delayed neuropathy (clinically or electromyographically confirmed), cognitive impairment (new-onset delirium or encephalopathy), and prolonged intensive care unit stay (>7 days).

### 2.8 Missing data handling

All candidate predictors and the primary outcome required for model development were fully observed in this cohort; therefore, no imputation procedures were applied.

### 2.9 Feature selection and model development

In the training cohort (n = 96), we used least absolute shrinkage and selection operator (LASSO) logistic regression to select an informative subset of predictors while mitigating multicollinearity and overfitting. The penalty parameter (lambda) was tuned by 10-fold cross-validation, and predictors with nonzero coefficients at the one-standard-error (1-SE) lambda were retained. Retained predictors were entered into a multivariable logistic regression model. Model refinement used backward stepwise selection (entry *p* < 0.05, removal *p* > 0.10) to derive the final parsimonious model. Treatment-related variables were considered exploratory predictors. However, given their potential dependence on baseline severity, their inclusion was carefully evaluated during penalized selection, and only variables demonstrating independent predictive value were retained in the final model.

### 2.10 Nomogram construction and model performance evaluation

Regression coefficients from the final multivariable model were used to construct a graphical nomogram. Discrimination was assessed by the area under the receiver operating characteristic curve (AUC) in both the training and validation cohorts. The optimal probability cutoff was chosen by maximizing Youden’s index in the training cohort.

Calibration was evaluated using calibration plots and mean absolute error derived from 500 bootstrap resamples. Clinical utility was quantified by decision curve analysis, plotting net benefit across threshold probabilities and comparing the nomogram against treat-all and treat-none strategies. In our context, treat-all denotes escalation of OP therapy (additional atropine boluses, continuous PAM infusion) and intensive care unit admission for all patients, whereas treat-none denotes management with standard supportive care on the general ward without proactive dose escalation or intensive monitoring.

### 2.11 Statistical analysis

Continuous variables are presented as mean ± standard deviation or median (interquartile range) and compared using Student’s *t*-test or the Mann–Whitney *U* test, as appropriate. Categorical variables are reported as counts (percentages) and compared using the chi-square test or Fisher’s exact test. LASSO regression was performed with the glmnet package; multivariable modeling, nomogram construction, calibration, and decision curve analysis were conducted in R (version 4.2.0). All tests were two-sided, and *p* < 0.05 was considered statistically significant.

## 3 Results

### 3.1 Baseline characteristics

A total of 138 patients with acute organophosphate poisoning were enrolled and randomly assigned to a training cohort (*n* = 96) and a validation cohort (n = 42). Baseline characteristics were well balanced between the two cohorts. The overall mean age was 39.09 ± 11.13 years (training 38.42 ± 11.84 vs. validation 40.64 ± 9.25; *p* = 0.237). Median baseline PSS scores were comparable [overall 13.0 (IQR 10.0–14.0); training 13.0 (10.0–14.0) vs. validation 12.5 (9.0–14.0); *p* = 0.330], as was the time from exposure to hospital admission [overall 4.0 h (1.63–8.02); training 4.30 h (2.08–8.40) vs. validation 2.95 h (0.43–6.70); *p* = 0.091]. Oral ingestion was the predominant exposure route (67.4%), followed by dermal contact (21.7%) and inhalation (10.9%), with no intergroup difference (*p* = 0.920). Laboratory and clinical indices at presentation also showed no significant between-cohort differences. Mean serum cholinesterase activity was 2,938 ± 1,035 U/L (training 3,000 ± 1,059 vs. validation 2,795 ± 974; *p* = 0.271), median GCS score was 13.0 (IQR 12.0–14.75; *p* = 0.422), mean lactate was 3.09 ± 0.97 mmol/L (*p* = 0.285), and arterial blood pH was 7.31 ± 0.11 (*p* = 0.405). Treatment-related variables were likewise similar: time to first atropine dose [median 1.13 h (0.40–1.96); *p* = 0.686], cumulative 24 h atropine dose (61.41 ± 28.25 mg; *p* = 0.692), and time to first pralidoxime (PAM) dose [median 1.51 h (0.66–2.64); *p* = 0.156]. The proportions of patients requiring mechanical ventilation (21.7%; *p* = 0.777) and those with major comorbidities (26.8%; *p* = 0.751) did not differ between cohorts (Table 1). The specific organophosphate agent was identified in 75 of 138 patients (54.3%). The most frequently documented agents were dichlorvos, chlorpyrifos, omethoate, and dimethoate (Supplementary Table S3).

**Table 1:** Baseline characteristics of patients with acute organophosphate poisoning stratified by training and validation cohorts.

Characteristics	Overall *N* = 138	Validation *N* = 42	Training *N* = 96	*p* value
Age, years	39.09 (11.13)	40.64 (9.25)	38.42 (11.84)	0.237^a^
Baseline PSS score	13.00 [10.00;14.00]	12.50 [9.00;14.00]	13.00 [10.00;14.00]	0.330^b^
Time to hospital admission, h	4.00 [1.63;8.02]	2.95 [0.43;6.70]	4.30 [2.08;8.40]	0.091^b^
Estimated dose, mL	48.74 (20.88)	45.44 (18.06)	50.19 (21.93)	0.187^a^
Exposure route				0.920^d^
Dermal	30 (21.74%)	10 (23.81%)	20 (20.83%)	
Inhalation	15 (10.87%)	4 (9.52%)	11 (11.46%)	
Oral	93 (67.39%)	28 (66.67%)	65 (67.71%)	
Serum cholinesterase, U/L	2,938 (1035)	2,795 (974)	3,000 (1059)	0.271^a^
GCS score	13.00 [12.00;14.75]	13.00 [12.00;14.75]	13.00 [12.00;14.25]	0.422^b^
Arterial lactate, mmol/L	3.09 (0.97)	3.23 (1.02)	3.03 (0.94)	0.285^a^
Arterial blood pH	7.31 (0.11)	7.32 (0.11)	7.31 (0.11)	0.405^a^
Time to first atropine, h	1.13 [0.40;1.96]	1.19 [0.49;1.92]	1.11 [0.40;1.98]	0.686^b^
Atropine dose in first 24 h, mg	61.41 (28.25)	59.85 (31.96)	62.10 (26.62)	0.692^a^
Time to first PAM, h	1.51 [0.66;2.64]	1.71 [0.98;2.85]	1.34 [0.50;2.59]	0.156^b^
Mechanical ventilation				0.777^c^
No	108 (78.26%)	34 (80.95%)	74 (77.08%)	
Yes	30 (21.74%)	8 (19.05%)	22 (22.92%)	
Major comorbidity				0.751^c^
No	101 (73.19%)	32 (76.19%)	69 (71.88%)	
Yes	37 (26.81%)	10 (23.81%)	27 (28.12%)	
Occupation				0.618^c^
Farmer	58 (42.0%)	16 (38.1%)	42 (43.8%)	
Industrial worker	40 (29.0%)	14 (33.3%)	26 (27.1%)	
Other	40 (29.0%)	12 (28.6%)	28 (29.2%)	
Residence				0.745^c^
Rural	85 (61.6%)	25 (59.5%)	60 (62.5%)	
Urban	53 (38.4%)	17 (40.5%)	36 (37.5%)	
Poisoning Intent				0.212^d^
Suicidal	70 (50.7%)	25 (59.5%)	45 (46.9%)	
Accidental	58 (42.0%)	14 (33.3%)	44 (45.8%)	
Homicidal	10 (7.3%)	3 (7.1%)	7 (7.3%)	
Gastric lavage				0.093^c^
Yes	46 (33.3%)	18 (42.9%)	28 (29.2%)	
No	92 (66.7%)	24 (57.1%)	68 (70.8%)	

PSS, poisoning symptom score; GCS, Glasgow Coma Scale; PAM, pralidoxime; ChE, cholinesterase; IQR, interquartile range; SD, standard deviation; MV, mechanical ventilation. Statistical tests: ^a^Student’s *t*-test; ^b^Mann–Whitney U test; ^c^Chi-square test; ^d^Fisher’s exact test; *p < 0.05. For clinical interpretability, reference values/ranges are as follows: normal consciousness corresponds to GCS = 15 (total score range 3–15); arterial lactate, approximately 0.5–2.2 mmol/L; arterial blood pH, 7.35–7.45; serum cholinesterase, a typical adult reference interval is approximately 5,300–12,900 U/L, although this is assay-dependent.

### 3.2 Feature selection by LASSO

**Figure 1:**
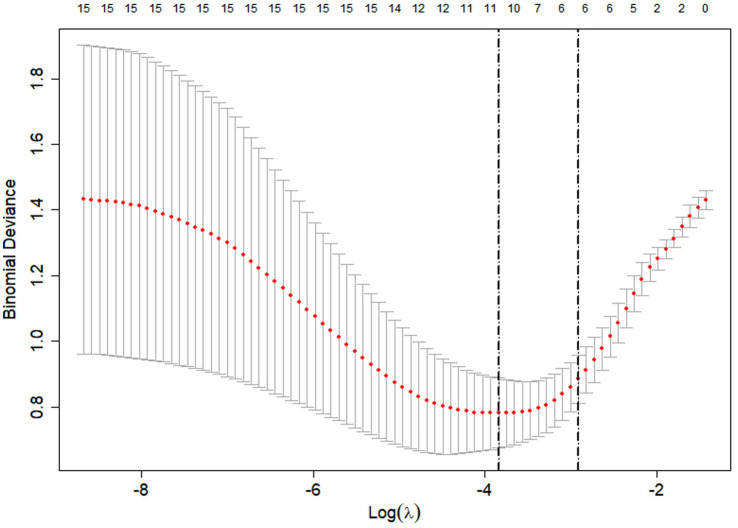
Ten-fold cross-validated binomial deviance plotted against log (*λ*), with the minimum-deviance *λ* (*λ*_min_) and the one-standard-error *λ* (*λ*_1se_) indicated by dashed lines.

**Figure 2:**
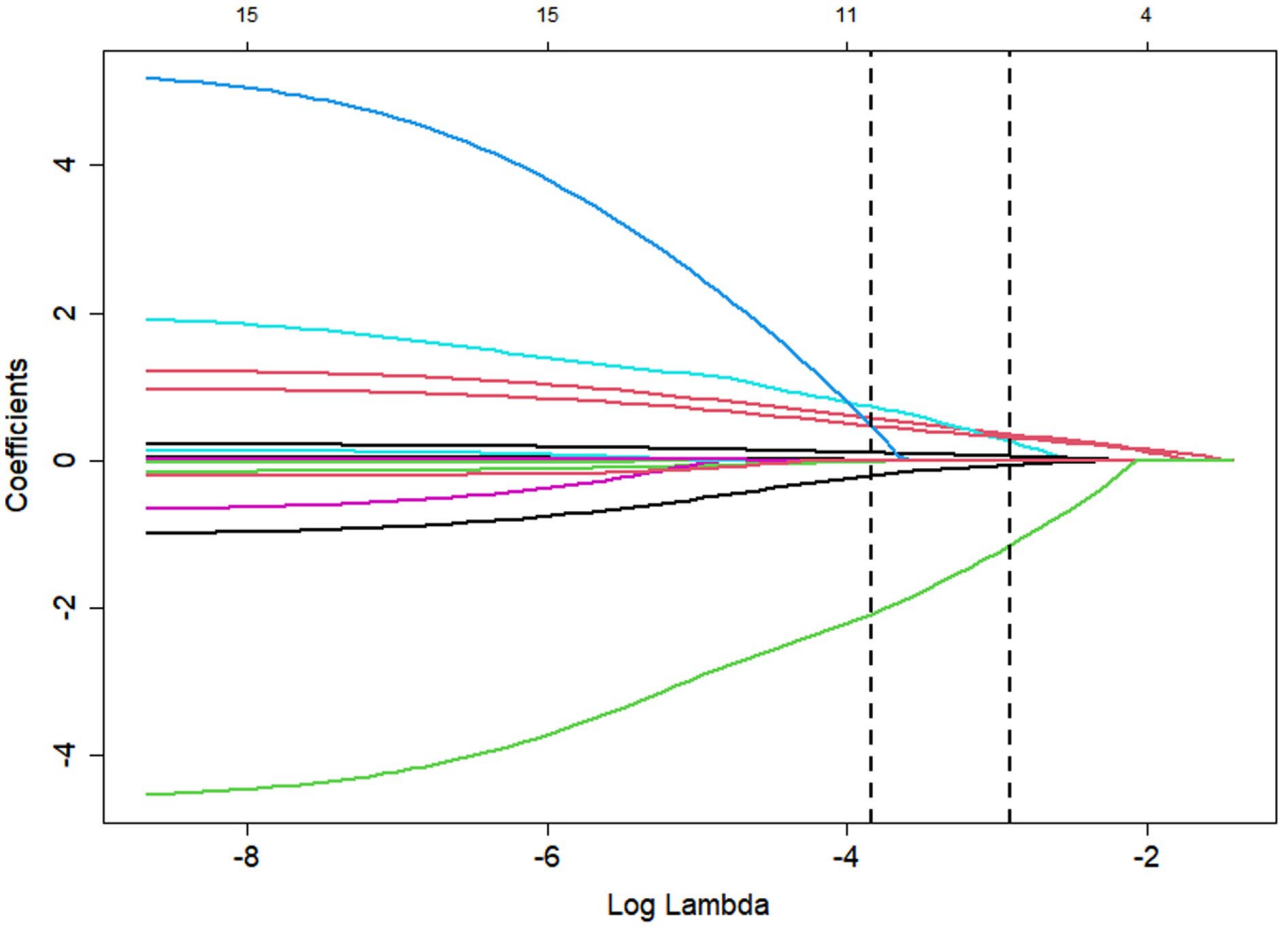
LASSO coefficient profiles for all 14 candidate predictors as a function of log (*λ*), highlighting the six nonzero coefficients retained at *λ*_1SE_.

To identify a parsimonious predictor set, we fitted a LASSO-penalized logistic regression to all 14 candidates and tuned the penalty by 10-fold cross-validation. Figure 1 shows the cross-validated binomial deviance with ±1-SE error bars. The left dashed line marks the penalty with the lowest error (log *λ*_min), and the right dashed line indicates the 1-SE rule, a slightly stronger penalty that favors parsimony with comparable error. The numbers above the panel give the count of non-zero coefficients at each penalty; at log *λ*₁ₛₑ, six predictors remained non-zero. Figure 2 (coefficient paths) illustrates how increasing the penalty shrinks coefficients toward zero; curves that touch zero are excluded. At log *λ*₁ₛₑ, the six retained predictors were: baseline PSS, exposure route (inhalation), serum cholinesterase, admission GCS, delay to first pralidoxime (PAM) dose, and major comorbidity.

### 3.3 Multivariable logistic regression analysis

All six predictors selected by LASSO [baseline PSS, exposure route, serum cholinesterase, GCS score, pralidoxime (PAM) delay, and comorbidity] were entered into a multivariable logistic regression model (Table 2). After adjustment, baseline PSS score remained a strong independent predictor of short-term symptom resolution (OR 2.81, 95% CI 1.80–5.29; *p* < 0.001), indicating that each one-point increase in initial PSS was associated with a 2.8-fold higher odds of remission. Serum cholinesterase activity was also independently associated with remission (OR 1.25 per 100 U/L increase, 95% CI 1.12–1.44; *p* < 0.001). GCS score on admission conferred a 2.23-fold higher odds of remission for each additional point (95% CI 1.33–4.27; *p* = 0.005). Presence of major comorbidities markedly reduced the odds of remission (OR 0.02, 95% CI 0.001–0.18; *p* = 0.001).

**Table 2:** Multivariable logistic regression for short-term symptom relief.

Characteristic	*B*	SE	OR	CI	*Z*	*p*
(Intercept)	−27.59	7.08849	**—**	**—**	−3.892	<0.001^*^
Baseline PSS score	1.033	0.2682	2.809	1.803–5.286	3.851	<0.001^*^
Serum cholinesterase (per 100 U/L)	0.221	0.0617	1.247	1.124–1.438	3.574	<0.001^*^
GCS score	0.802	0.28881	2.231	1.334–4.271	2.778	0.005^*^
Time to first PAM, h	−0.622	0.35493	0.537	0.248–1.009	−1.753	0.08
Major comorbidity (yes vs. no)	−3.747	1.16857	0.024	0.001–0.180	−3.206	0.001^*^
Exposure route: inhalation vs. dermal	2.431	2.70894	11.373	0.195–2292.0	0.897	0.369
Exposure route: oral vs. dermal	0.414	0.868	1.513	0.280–9.086	0.477	0.633

B, regression coefficient; SE, standard error; OR, odds ratio; CI, confidence interval; PSS, poisoning symptom score; GCS, Glasgow Coma Scale; PAM, pralidoxime; ChE, cholinesterase. Statistical test: *e* Wald *z* test (two-sided). *p < 0.05.

### 3.4 Development of a nomogram for short-term remission prediction

**Figure 3:**
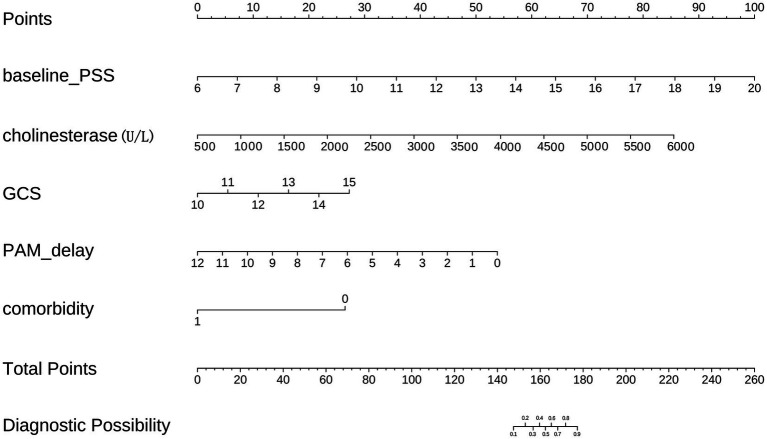
Nomogram for predicting the probability of short-term symptom relief after organophosphate poisoning. Variables are scored on the “Points” scale, and the sum of the four predictor-specific point values maps to the predicted probability of symptom relief. The nomogram incorporates baseline PSS score, serum cholinesterase activity, admission GCS score, and major comorbidity status.

A nomogram was constructed from the final four-variable logistic regression model to provide a simple bedside tool for estimating the probability of short-term symptom relief (Figure 3). The final nomogram incorporated baseline PSS score, serum cholinesterase activity, admission GCS score, and major comorbidity status. To use the nomogram, the clinician locates each patient’s value on the corresponding predictor axis, reads off the assigned points, sums the four point totals, and then projects the total to the probability scale to obtain the estimated likelihood of 48-h symptom resolution. Supplementary Table S1 lists the final logistic regression coefficients and overall model fit statistics used to construct the nomogram.

### 3.5 Discriminative performance of the prediction model

**Figure 4:**
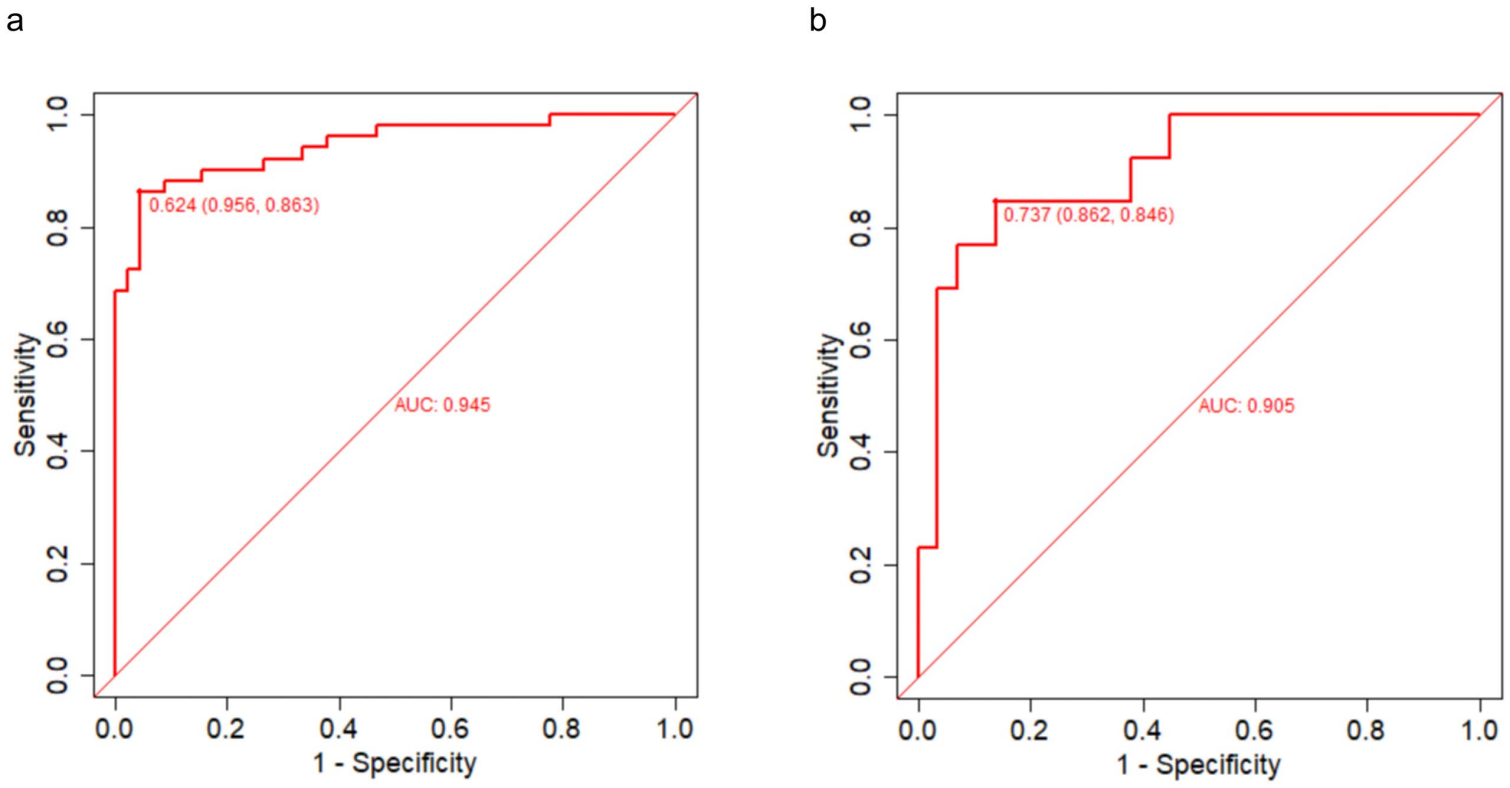
Receiver operating characteristic (ROC) curves of the nomogram in the training and validation cohorts. **(a)** Training cohort; **(b)** validation cohort. The ROC curves show the discriminatory performance of the nomogram for predicting 48-h symptom resolution (ΔPSS >0), with corresponding AUC values displayed in each panel.

The discriminative performance of the four-variable nomogram was first assessed in the training cohort (*n* = 96) by ROC analysis (Figure 4a). The area under the curve (AUC) was 0.945, indicating excellent separation between patients who did versus did not achieve short-term symptom resolution. After bootstrap optimism correction (*B* = 1,000), the AUC was 0.930. At the Youden-optimal cutoff probability of 0.624, sensitivity and specificity were 95.6 and 86.3%, respectively (Figure 4a). In the independent validation cohort (*n* = 42), internal validation confirmed robust discrimination with an AUC of 0.905 (Figure 4b). Using a cutoff of 0.737, sensitivity was 86.2% and specificity 84.6%. These results demonstrate that the nomogram maintained strong discriminative ability across both the training and validation cohorts.

### 3.6 Calibration of the nomogram

**Figure 5:**
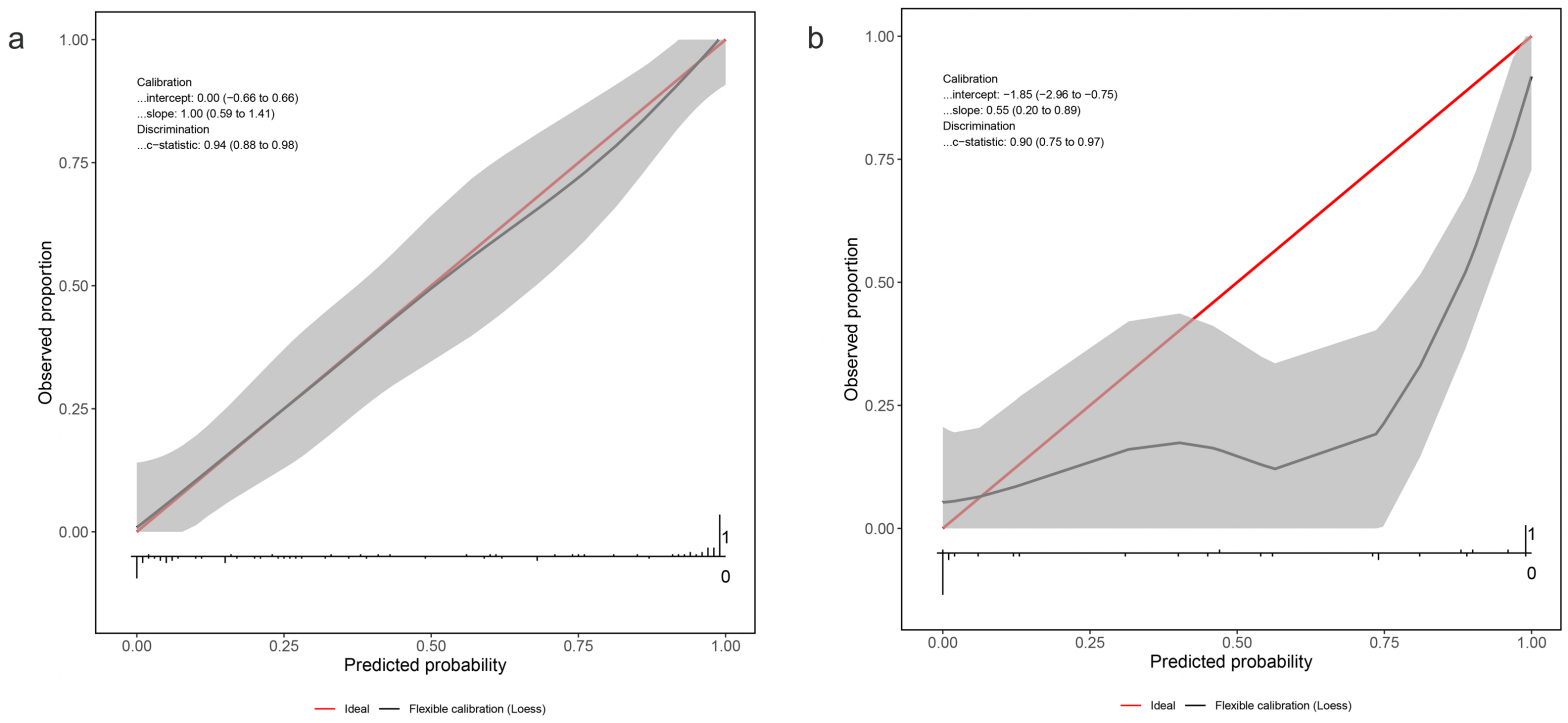
Calibration plots of the nomogram in the training and validation cohorts. **(a)** Training cohort; **(b)** Validation cohort. The red diagonal line indicates ideal calibration. The black curve shows flexible calibration. The shaded region denotes the 95% confidence interval for the calibration curve. Rug plots along the *x*-axis show the distribution of predicted probabilities. Calibration intercept/slope and discrimination (*C*-statistic) are annotated in each panel.

Calibration was assessed using bootstrap-corrected plots in both the training and validation cohorts. In the training cohort (*n* = 96; Figure 5a), the logistic calibration curve (solid black) nearly overlapped the 45° ideal line (grey), and the nonparametric curve (dotted) showed only minimal deviation. The bootstrap mean absolute error (500 resamples) was 0.034, reflecting excellent internal calibration (intercept = 0.00, slope = 1.00). In the validation cohort (*n* = 42; Figure 5b), calibration remained acceptable for mid-range probabilities (0.2–0.8), although the model slightly underestimated high remission probabilities (predicted >0.8), with a validation intercept of −1.20, slope of 0.547, and MAE of 0.066. Accordingly, we applied uniform shrinkage using the validation slope (*s* = 0.547) to all non-intercept coefficients and re-estimated the intercept to correct overestimation; the resulting shrunken coefficients and recalibrated intercept (α* = −15.917) are shown in Supplementary Table S2.

### 3.7 Decision curve analysis

**Figure 6:**
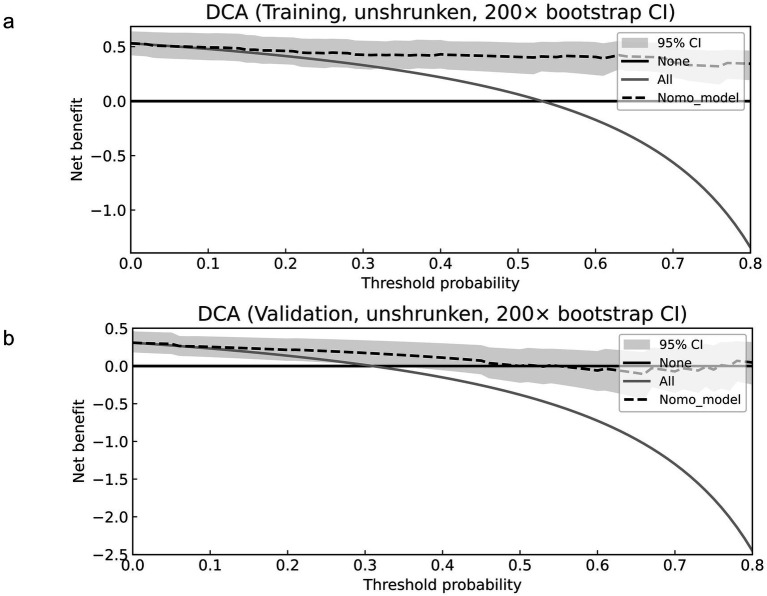
Decision curve analysis (DCA) of the nomogram in the training and validation cohorts. **(a)** Training cohort; **(b)** validation cohort. The dashed line represents the net benefit of the nomogram-based strategy across threshold probabilities. The “treat-all” and “treat-none” strategies are shown for comparison. Shaded bands indicate 95% bootstrap confidence intervals (200 resamples). Higher net benefit across a clinically plausible range of thresholds indicates greater potential clinical utility of the nomogram.

Decision-curve analysis showed that, across a broad range of threshold probabilities, the model provided greater net benefit than both treat-all and treat-none strategies in the training cohort (*n* = 96; Figure 6a) and the validation cohort (*n* = 42; Figure 6b). In the training cohort, gains were observed from approximately 0.05 to 0.80; for example, at a 20% threshold, the model net benefit was 0.461 (95% CI 0.344–0.581) versus 0.414 for treat-all and 0 for treat-none, corresponding to about 18.8 fewer unnecessary interventions per 100 patients. At a 10% threshold, the net benefit was 0.493 (95% CI 0.382–0.617) versus 0.479 for treat-all, corresponding to about 12.5 fewer unnecessary interventions per 100 patients. In the validation cohort, the model outperformed both comparators from approximately 0.05 to 0.60, with expected variability at very high thresholds (>0.70) because of the smaller sample size. At a 10% threshold, net benefit was 0.254 (95% CI 0.127–0.384) versus 0.233 for treat-all, corresponding to about 19.0 fewer unnecessary interventions per 100 patients; at a 20% threshold, net benefit was 0.214 (95% CI 0.077–0.357) versus 0.137, corresponding to about 31.0 fewer unnecessary interventions per 100 patients.

### 3.8 Complication patterns by 28-day survival status

Of the 138 patients, more than half (55.1%) experienced no complications, while 27.5% developed only short-term events, 4.3% only long-term events, and 13.0% both (Table 3). Stratified by 28-day survival, all 10 non-survivors had at least one complication: 3 (30.0%) had only short-term events, 1 (10.0%) only long-term events, and 6 (60.0%) both. By contrast, 76 of 128 survivors (59.4%) remained complication-free, 35 (27.3%) had only short-term events, 5 (3.9%) only long-term events, and 12 (9.4%) both (Table 3).

**Table 3:** Complication patterns by 28-day survival status.

Complication category	Overall (*n* = 138)	Survivors (*n* = 128)	Non-survivors (*n* = 10)
No complications	76 (55.1%)	76 (59.4%)	0 (0.0%)
Only short-term (≤48 h)	38 (27.5%)	35 (27.3%)	3 (30.0%)
Only long-term (3–28 d)	6 (4.3%)	5 (3.9%)	1 (10.0%)
Both short- and long-term	18 (13.0%)	12 (9.4%)	6 (60.0%)

h, hours; d, days.

### 3.9 Symptom incidence

On admission, the most frequent cholinergic and neuromuscular signs were sweating (75/138; 54.3%), vomiting (68/138; 49.3%), and chest crepitations (60/138; 43.5%). Diarrhea occurred in 54 patients (39.1%), abdominal colic in 50 (36.2%), fasciculations in 40 (29.0%), and muscle weakness in 30 (21.7%). There were no significant differences in any of these symptom rates between the training and validation cohorts (all *p* > 0.05).

## 4 Discussion

In this retrospective cohort study, we developed and internally validated a nomogram to estimate the probability of 48-h symptom resolution in patients with acute organophosphate (OP) poisoning, defined as an improvement in the poisoning symptom score (ΔPSS > 0). Using a training cohort of 96 patients and an internal validation cohort of 42 patients, LASSO regression first selected six candidate predictors from the initial variable set. After multivariable logistic regression and model refinement, four independent predictors remained in the final nomogram: baseline PSS score, serum cholinesterase activity on admission, admission Glasgow Coma Scale (GCS) score, and major comorbidity status. The final model demonstrated excellent discrimination (AUC 0.945 in the training cohort and 0.905 in the validation cohort) with acceptable calibration (mean absolute error 0.034 and 0.066, respectively). Decision curve analysis (DCA) suggested net clinical benefit across a wide range of threshold probabilities, supporting the potential utility of the nomogram as an early decision-support aid.

Our findings add to a growing literature on risk stratification in OP and pesticide poisoning, but with a distinct clinical target. Many recent prognostic models emphasize hard endpoints such as mortality, shock, or early respiratory failure, which are critical for triage but do not directly quantify short-term symptom trajectory during the first 48 h. For example, Sun et al. (19) developed a mortality-oriented model that combined age, acetylcholinesterase-related biomarkers, and AST to achieve high discrimination in a retrospective OP-poisoning cohort. In a broader acute pesticide-poisoning population, Wang et al. proposed a four-variable nomogram to predict early respiratory failure, emphasizing escalation decisions and including consciousness level as a key component (20). In contrast, our nomogram focuses on early symptom improvement (ΔPSS > 0), a pragmatic endpoint that may be more directly linked to bedside monitoring intensity and the expected pace of clinical stabilization. Taken together, the current literature suggests that complementary models may be needed for different decisions (e.g., ICU triage vs. expected early recovery), and our work provides a step toward individualized estimation of early symptom resolution.

Baseline PSS score was the strongest contributor to the nomogram, consistent with the concept that global symptom burden at presentation reflects the early physiologic impact of OP intoxication. In the present study, the PSS summarized five domains—gastrointestinal, respiratory, cardiovascular, central nervous system, and metabolic/general manifestations—each graded from 0 to 4 according to the most severe documented findings, for a total score range of 0–20. A higher baseline PSS therefore indicates broader or more severe multi-system involvement at admission. Because our endpoint was defined as any improvement in the score over 48 h (ΔPSS > 0), baseline severity can also influence the probability of observing measurable short-term change; nonetheless, a high baseline PSS remains clinically meaningful as a marker of acute risk and should continue to prompt vigilant monitoring and timely supportive care.

Serum ChE activity on admission emerged as a robust predictor of short-term symptom resolution, reinforcing its role as a quantitative surrogate of cholinesterase inhibition and exposure intensity. We observed that each 100 U/L increase in ChE activity corresponded to an approximately 25% increase in the odds of symptom resolution within 48 h (OR 1.25, 95% CI 1.12–1.44; *p* < 0.001). Consistent with this, prior studies have linked low cholinesterase activity to more severe poisoning, increased ventilatory requirements, and higher mortality (21). Serial monitoring may add additional prognostic value by capturing recovery kinetics; faster ChE normalization has been associated with shorter ICU stays and improved trajectories (22). Notably, recent predictive work aimed at mortality also retained cholinesterase-related biomarkers as key components alongside systemic injury markers, emphasizing the broader prognostic relevance of cholinesterase inhibition beyond symptom scoring alone (19).

The GCS score on admission independently predicted 48-h symptom remission, highlighting the importance of early neurologic assessment and central cholinergic involvement. We found that each one-point increase in GCS increased the odds of 48-h symptom remission by approximately 2.2-fold (OR 2.23, 95% CI 1.33–4.27; *p* = 0.005). This is compatible with previous observations that depressed consciousness is a major driver of early respiratory complications; for instance, Sam et al. (6) identified a GCS ≤ 8 threshold associated with markedly higher risk of respiratory failure. A composite score integrating GCS and laboratory indices has also been shown to improve prediction of ventilatory support needs (23). Importantly, the central role of consciousness level is reinforced by recent nomogram work for early respiratory failure in acute pesticide poisoning, in which a GCS < 15 was retained as one of four core predictors (20).

Delay in PAM administration showed a clinically meaningful negative trend for early symptom resolution, although it did not reach conventional statistical significance (OR 0.54, 95% CI 0.27–1.08; *p* = 0.08). This trend is biologically plausible, as treatment delays increase the likelihood of “aging” of the enzyme–OP complex, potentially reducing the efficacy of oxime-mediated reactivation. PAM delay did not remain statistically significant after multivariable adjustment, potentially reflecting confounding by indication, since patients with more severe poisoning are more likely to receive earlier and higher-intensity treatment. Nevertheless, prior studies support the clinical relevance of early oxime therapy: in a multicenter retrospective study, Eddleston et al. (24) reported lower mortality with early PAM administration compared with delayed use, and a prospective Indian trial suggested that earlier oxime therapy shortened time to cholinesterase recovery and reduced the incidence of intermediate syndrome (25). Furthermore, prospective observational data indicate that delayed presentation to care is associated with substantially higher fatality (20), which aligns with the broader principle that timeliness of antidotal and supportive therapy influences early physiologic stabilization. Although our study may have been underpowered to detect a statistically significant PAM-delay effect, the observed direction and supporting literature underscore early oxime consideration as a potentially modifiable factor in acute OP poisoning.

Comorbidity status markedly decreased the likelihood of rapid symptom resolution (OR 0.03, 95% CI 0.004–0.19; *p* = 0.001). Preexisting cardiovascular disease, chronic liver or renal impairment, and diabetes can reduce organ reserve, alter toxicokinetics and drug handling, and complicate atropine titration or ventilatory management. Consistent with our findings, Yu et al. (26) reported substantially higher mortality risk among elderly OP-poisoned patients with multimorbidity, and Li et al. (27) described prolonged cholinesterase inhibition and longer ventilatory support among patients with chronic liver disease. These data underscore that baseline health status should be explicitly incorporated into early prognostic assessment and escalation planning.

Although the route of exposure is an important determinant of OP toxicokinetics, it did not remain in the final model after penalized selection and multivariable adjustment. Inhalational exposure may yield rapid absorption and higher early peak concentrations, whereas dermal exposure can produce delayed and prolonged toxicity (28). In our cohort, exposure-route heterogeneity and the retrospective nature of ascertainment may have reduced measurement precision, thereby attenuating its independent predictive contribution. Future studies should examine compound-specific effects, exposure route, and dose proxies in multicenter datasets to determine whether route meaningfully improves prediction beyond bedside severity and early physiologic measures.

From a methodological perspective, LASSO-assisted feature selection helped address overfitting risk and multicollinearity while preserving interpretability of the final logistic model. We evaluated model performance using discrimination, calibration, and net benefit, consistent with best practice in prognostic modeling (29, 30). Given the rapid evolution of reporting and appraisal standards, mapping our model development and evaluation to the TRIPOD+AI checklist (which supersedes TRIPOD 2015) could further strengthen transparency and reproducibility, particularly around data handling, predictor measurement, and internal validation strategy (31). In addition, use of PROBAST+AI would enable a structured assessment of potential risk of bias and applicability issues for regression-based or machine-learning models alike (32). The nomogram format remains a pragmatic advantage, allowing bedside estimation without specialized software; however, integration into an electronic health record (EHR) calculator or mobile tool would improve usability and reduce transcription error.

Several strengths warrant emphasis: (i) predictors are readily available during initial assessment; (ii) the model achieved high discrimination in both training and internal validation samples; (iii) calibration and DCA were explicitly reported; and (iv) internal validation included bootstrap-based calibration assessment to mitigate optimism. Despite these strengths, several limitations should be acknowledged. First, the single-center, retrospective nature of this study may limit the generalizability of our findings and introduce selection bias. Second, analytical variability in cholinesterase (ChE) assays across laboratories could impact the model’s portability. Additionally, emerging biomarkers, such as paraoxonase-1 (PON1) activity and butyrylcholinesterase genotype, which have shown prognostic relevance, were not included in our model. Third, while we performed internal validation using bootstrap correction, the lack of external validation remains a key limitation, and future studies should aim for multicenter external validation with site-specific recalibration to assess model transportability. Finally, the comorbidity variable, though practical, serves as a surrogate for the Charlson Comorbidity Index and may misclassify multimorbidity, reflecting limited granularity due to small cell counts.

In clinical practice, this nomogram can assist in early risk stratification, optimize ICU resource allocation, and guide discussions with patients and their families. Patients predicted to have a low probability of 48-h symptom resolution may benefit from closer monitoring and earlier intervention, while those predicted to improve rapidly might avoid unnecessary ICU admission. However, prospective multicenter studies are needed to validate this tool externally and to explore whether extending its predictive horizon to intermediate syndrome or delayed neuropathy adds clinical value. Future work should also investigate the integration of additional biomarkers and the development of a digital version for enhanced usability in clinical settings.

## 5 Conclusion

In conclusion, we developed a parsimonious, clinically intuitive nomogram to estimate 48-h symptom resolution after acute organophosphate poisoning. Discrimination was strong and internal calibration acceptable, but an internal-validation calibration slope of 0.547 indicates risk overestimation; accordingly, we provide uniformly shrunken non-intercept coefficients and a recalibrated intercept to support transparent external testing. Decision-curve analysis suggests potential clinical utility, yet these gains are threshold- and calibration-dependent. Clinical adoption should await prospective multicenter external validation with site-specific recalibration as needed, alongside inter-observer reliability assessment for PSS/GCS and clinical anchoring of ΔPSS to hard outcomes; biomarker augmentation and digital deployment will be explored thereafter.

## Data Availability

The raw data supporting the conclusions of this article will be made available by the authors, without undue reservation.
