# Supplementary material for: Development and validation of a predictive model for short-term symptom relief after organophosphate poisoning
Source: Front Med (Lausanne). 2026 Apr 28;13:1637549. doi: 10.3389/fmed.2026.1637549 (PMC13160850; doi:10.3389/fmed.2026.1637549)
Supplement: Supplementary file 1 [file Table_1.docx]

**Table S1. Four-Variable Logistic Regression Model Coefficients and Fit Statistics**

| Metric | Value |
| --- | --- |
| Intercept (β) | -26.9011 |
| Baseline PSS score (β) | 1.0391 |
| Serum cholinesterase (per 100 U/L) (β) | 0.1987 |
| GCS score (β) | 0.7412 |
| Major comorbidity (β) | -4.0336 |
| Null df | 95 |
| Residual df | 91 |
| Null deviance | 132.7 |
| Residual deviance | 56.42 |
| AIC | 66.42 |

Abbreviations: **β**, regression coefficient (log-odds); **AIC**, Akaike information criterion; **df**, degrees of freedom.

**Table S2. Shrunken coefficients using the validation calibration slope (s = 0.547)**

| Predictor | Original β | Shrunken β (=0.547×β) | Original OR | Shrunken OR (=exp(0.547×β)) |
| --- | --- | --- | --- | --- |
| Baseline PSS score | 1.039 | 0.568 | 2.827 | 1.765 |
| Serum cholinesterase (per 100 U/L) | 0.199 | 0.109 | 1.22 | 1.115 |
| GCS score | 0.741 | 0.405 | 2.098 | 1.5 |
| Major comorbidity | -4.034 | -2.207 | 0.018 | 0.11 |
| Intercept | -26.901 | α = -15.917* |  |  |

Abbreviations: **β**, regression coefficient (log-odds); **OR**, odds ratio; **exp**, exponential function.

**Table S3. Distribution of identifiable organophosphate agents among patients with available poison-name records**

| Agent | Chemical subgroup | n | % among identifiable cases |
| --- | --- | --- | --- |
| Dichlorvos | Dimethyl OP | 16 | 21.3 |
| Chlorpyrifos | Diethyl OP | 12 | 16 |
| Omethoate | Dimethyl OP | 10 | 13.3 |
| Dimethoate | Dimethyl OP | 9 | 12 |
| Phorate | Diethyl OP | 7 | 9.3 |
| Methamidophos | Dimethyl OP | 5 | 6.7 |
| Triazophos | Diethyl OP | 5 | 6.7 |
| Monocrotophos | Dimethyl OP | 4 | 5.3 |
| Methyl parathion | Dimethyl OP | 3 | 4 |
| Quinalphos | Diethyl OP | 2 | 2.7 |
| Diazinon | Diethyl OP | 2 | 2.7 |
| Total |  | 75 | 100 |
